# Iron sulfate and molasses treated anthocyanin-rich black cane silage improves growth performance, rumen fermentation, antioxidant status, and meat tenderness in goats

**DOI:** 10.5713/ab.22.0252

**Published:** 2022-09-07

**Authors:** Rayudika Aprilia Patindra Purba, Ngo Thi Minh Suong, Siwaporn Paengkoum, Pramote Paengkoum, Juan Boo Liang

**Affiliations:** 1 School of Animal Technology and Innovation, Institute of Agricultural Technology, Suranaree University of Technology, Muang 30000, Thailand; 2 School of Animal Sciences, Agriculture Department, Can Tho University, Can Tho City 92000, Vietnam; 3 Program in Agriculture, Faculty of Science and Technology, Nakhon Ratchasima Rajabhat University, Muang 30000, Thailand; 4 Laboratory of Sustainable Animal Production and Biodiversity, Institute of Tropical Agriculture and Food Security, University Putra Malaysia, Serdang 43400, Malaysia

**Keywords:** Agricultural Waste, Anthocyanin, Antioxidant Capacity, Carcass Characteristics, Iron-Treated Silage, Molasses-treated Silage

## Abstract

**Objective:**

This study investigated the effects of feeding anthocyanin-rich black cane treated with ferrous sulfate and molasses on animal performance, rumen fermentation, microbial composition, blood biochemical indices, and carcass characteristics in meat goats.

**Methods:**

Thirty-two Thai-native×Anglo-Nubian crossbred male goats (14.47±2.3 kg) were divided equally into two groups (n = 16) to investigate the effect of feeding diet containing 50% untreated anthocyanin-rich black cane silage (BS) vs diet containing anthocyanin-rich black cane silage treated with 0.03% ferrous sulfate and 4% molasses (TBS) on average daily gain (ADG) and dry matter intake (DMI). At the end of 90 d feeding trial, the goats were slaughtered to determine blood biochemical indices, rumen fermentation, microbial composition, and carcass characteristics differences between the two dietary groups.

**Results:**

Goats fed the TBS diet had greater ADG and ADG to DMI ratio (p<0.05). TBS diet did not affect rumen fluid pH; however, goats in the TBS group had lower rumen ammonia N levels (p<0.05) and higher total volatile fatty acid concentrations (p<0.05). Goats in the TBS group had a higher (p<0.05) concentration of *Ruminococcus albus* but a lower (p<0.05) concentration of methanogenic bacteria. The TBS diet also resulted in lower (p<0.05) thiobarbituric acid-reactive substances concentration but higher (p<0.05) total antioxidant capacity, superoxide dismutase, catalase, glutathione peroxidase, and glutathione reductase concentrations in blood plasma, while having no effect on plasma protein, glucose, lipid, immunoglobin G, alanine transaminase, and aspartate aminotransferase. Meat from goats fed the TBS diet contained more intramuscular fat (p<0.05) and was more tender (p<0.05).

**Conclusion:**

In comparison to goats fed a diet containing 50% untreated anthocyanin-rich black cane silage, feeding a diet containing 50% anthocyanin-rich black cane silage treated with 0.03% ferrous sulfate and 4% molasses improved rumen fermentation and reduced oxidative stress, resulting in higher growth and more tender meat.

## INTRODUCTION

The application of ferrous sulfate (FS) to enhance the nutritional value of lignocellulosic biomass during roughage preservation for ruminant feed is well established [1]. Incorporating FS during the ensiling process has the potential to accelerate the breakdown of biomass, leading to the formation of more functional microbes and compounds that lower nutrients loss and formation of toxic substances, consequently increasing the productivity in ruminants [1,2]. Several direct-fed FS studies have shown to increase the health and productivity of small ruminants [2,3]. Direct-fed FS method either administered FS orally in the form of a capsule or incorporating it into the diet [4]. More recently, study showed that addition of FS to molasses (MS) as a mixed additive (FS-MS) enhanced the effectiveness of the ensiling process and goat performance [5].

Heat-induced oxidative stress is a well-known limiting factor affecting animal health and performance in the tropics [6,7], and feeding anthocyanin-rich black cane (*Saccharum sinensis* Robx.) to tropical ruminants, including slow-growing indigenous goats, can be a practical approach to overcome the above production constraint [8]. In Thailand, anthocyanin-rich black cane (hybrid of *Saccharum spontaneum* and *Saccharum officinarum*) was recently recognized as a potential alternative method for sugar production, while the leftover biomass (stalks and leaves), including in the form of silage, could be used as ruminant feed [1,5]. It is possible that, in the tropics, feeding diet containing anthocyanin to goats is beneficial because anthocyanin has the potential to enhance the antioxidant capacity of the rumen [8] which in turn reduces oxidative stress in the animals by limiting the availability of free radicals and the generation of additional oxidations [6,8]. However, the highly lignified components of anthocyanin-rich black cane limit anthocyanin bio-accessibility during feeding. Thus, incorporating FS-MS mixture to anthocyanin-rich black cane prior to ensiling can be useful. Earlier studies revealed that treating anthocyanin-rich black cane with FS-MS mixture (0.15% to 0.03% FS and 4% to 8% MS on fresh weight silage basis) decreased lignin contents, improved anthocyanin stability, and *in vitro* ruminal fermentation of black cane silage [1,5]. Recent *in vivo* studies reported that the microbial population in rumen fluid of goats fed purple corn [9] and black cane [8] containing anthocyanin enhanced the acetate to propionate ratio. The anthocyanin-induced acetic acid production may result in more substrate available for *de-novo* fat synthesis [10], however, because the molecular structure of anthocyanins varied depending on the plant species or during processing, it is difficult to accurately generalize the earlier findings [8,9].

As far as we know, evaluation of FS-MS treated anthocy anin-rich black cane silage as ruminant feed has been limited only to laboratory scale silos and *in vitro* procedures [1,5]. Therefore, the present study investigated the effects of feeding a standard total mixed ration (TMR) containing anthocyanin-rich black cane silage treated with FS-MS mixture on animal performance, rumen fermentation, microbial community, blood biochemical indices, and carcass characteristics in meat goats.

## MATERIALS AND METHODS

All procedures described herein were supervised and approved by the Animal Ethics Committee of Suranaree University of Technology (SUT 4/2558). The research was conducted in accordance with regulations on animal experimentation and the Guidelines for the Use of Animals in Research, as recommended by the National Research Council of Thailand (U1-02632-2559).

### Roughage harvesting and ensiling

Anthocyanin-rich black cane was cultivated from a goat and sheep research farm in Suranaree University of Technology, Nakhon Ratchasima Province, Thailand (14°52′49.1″N, 102°00′14.9″E at an elevation of 243 m above sea level). The field study was conducted between August 2018 and February 2019. The management of forage including the use of fertilizer was as previously reported [1]. On the 180th d after initial planting, fresh anthocyanin-rich black cane was randomly sampled from six locations (n = 6) by cutting approximately 10 cm above ground level [8]. The collected materials (stalks and leaves) were then mechanically chopped to approximately 2 to 3 cm using a crop cutter (7.5 HP, SF-JR, Mitsubishi electric automation, Bangkok, Thailand) and thoroughly mixed. The chopped anthocyanin-rich black canes were ensiled without wilting following two treatments: i) anthocyanin-rich black cane only (BS) and ii) anthocyanin-rich black cane treated with FS heptahydrate (FeSO_4_×H_2_O; Merck KGaA, Darmstadt, Germany, 0.03%) and MS (4%, TBS) mixture on fresh matter basis. Ensiling procedure, including the preparation and incorporation of FS-MS into silages, was as described previously [1,5]. The prepared silages were stored in plastic drums (110 L; diameter, 464 mm; height, 750 mm; Misumi, Bangkok, Thailand) and stored at 25°C to 27°C. The above two silages were fermented for 90 d and the silages were shown to be of high quality as indicated by sensory evaluation and pH measurement (portable pH meter, Oakton pH 700; Long Branch, NJ, USA). The chemical composition and fermentation properties of the untreated (BS) and treated (TBS) anthocyanin-rich black cane silages are presented in Table 1. There were no differences among the different chemical components except the lignin content was approximately 9.1% lower for the TBS as compared to the untreated BS. The TBS sample had lower pH (3.7 vs 4.8) and higher lactic and acetic acids.

### Animals and dietary treatments

A total of 32 healthy Thai-native×Anglo-Nubian crossbred male goats with an initial average body weight (BW; 14.47± 2.3 kg) were used for this study. These goats were obtained from the SUT farm and nearby local farms. The goats were housed individually and randomly assigned to one of the following two experimental diets (Table 2): i) TMR contained 50% untreated silage (BS) diet as control (n = 16) or ii) TMR contained 50% treated silage (TBS) diet (n = 16). The remaining ingredients in the TMR were identical between the two diets which were formulated to meet the requirement of NRC [11]. The two diets which consisted of 50% silage (untreated vs treated) were iso-caloric and iso-nitrogenous. The fiber components of the two diets were nearly similar except for lignin which was about 10% higher for the TBS diet. The nutrient composition showed a 5-fold higher anthocyanin content in the TBS sample as compared with the untreated BS, which was primarily due to higher contents of the peonidin-3-O-glucoside, malvidin-3-O-glucoside, and malvidin fractions in the TBS sample (Table 2).

Goats were subjected to a 14-d adaptation period in which the experimental diets were gradually introduced to them prior to the 90-d feeding trial. The TMR was offered to the goats (at 0.95×the voluntary feed intake previously determined during the adaptation period) twice daily in equal proportions at 0700 and 1600. The goats had full access to clean drinking water and trace-minerals block. Daily feed refusals were recorded before morning feeding and used to determine dry matter intake (DMI). The BW of the goats was recorded weekly before morning feeding to determine growth performance during the feeding trial.

### Sample collection, and laboratory analysis

Samples of two experimental diets, and feed refusals were collected every two weeks, pooled, dried at 55°C in an air dry oven, and pulverized in a Wiley Mill equipped with a (Retsch SM 100 mill; Retsch Gmbh, Haan, Germany) with a 1-mm screen. The dried samples were analyzed for their chemical and nutritional composition. The nitrogen content of the experimental diets and diet refusals was determined using a KjeltecTM 8400 fully automated Kjeldahl analyser (FOSS, Hilleroed, Denmark) and a conversion factor of 6.25 was used to calculate the crude protein (CP) values. Neutral detergent fiber (NDF, with heat-stable α-amylase), acid detergent fiber (ADF), and acid detergent lignin (ADL) were determined using a fully automated method (Fibertec 800; FOSS, Denmark) according to AOAC methods [12]. Hemicellulose content was calculated by subtracting ADF from NDF and cellulose content was calculated by subtracting ADL from ADF. In addition, the supernatant of the second subsample of dried samples was transferred to a 50-mL volumetric flask for HPLC measurement of anthocyanin content after being extracted at 50°C for 24 h with 0.01 N hydrochloric acid (HCl) dissolved in 80% methanol [13]. The chromatographic separation was performed in triplicates using a reversed-phase Zorbax SB-C18 (3.5 μm particle size, i.d. 4.6 mm×250 mm; Agilent Technologies, Santa Clara, CA, USA) for 65 min at 28°C and evaluated using a photodiode array UV detector with a 520 nm wavelength setting [14,15]. Notably, when the measured nutrient values of the diet differed from the original values, the formula for the diet was modified to conform to the measured nutrient values.

A 10-mL heparin-containing vacuum tube was used to collect blood samples from the jugular vein 2 h after the last morning feeding during the last feeding week. Plasma concentrations of urea, total protein, glucose, insulin, triglycerides, alanine transaminase, and aspartate aminotransferase were determined in quadruplicate using an automated enzymatic colorimetric method on a Cobas Integra 400 Instrument (Roche Diagnostics, Mannheim, Germany) [8]. The remaining plasma was then used to assess plasma antioxidant (total antioxidant capacity [TAC], thiobarbituric acid-reactive substances [TBARS], superoxide dismutase [SOD], catalase [CAT], glutathione peroxidase [GSH-Px], and glutathione reductase [GSH-Rx]) levels on a Microplate (96 wells, UV plate), quadruplicate, equipped with a microreader (Varioskan-LUX multimode microplate reader, Thermo Scientific, USA), as previously described [6].

### Sampling and carcass measurements

At the completion of the 90-d feeding trial, all the 32 goats were slaughtered. Prior to slaughtering, the final BW of goats were taken and feed were withdrawn for 24 h before being transferred to the university’s slaughterhouse facility for slaughtering and collection of rumen fluid samples [16].

#### Rumen fluid

The composite rumen content sample from each slaughtered goat was then filtered through four layers of cheesecloth to extract rumen fluid. Using a portable pH meter, the pH of the rumen fluid was determined immediately. The rumen fluid was then transferred to the lab in a thermos flask that had been sterilized. Upon arrival at the laboratory, the filtered rumen fluid was divided into two aliquots. The first aliquot of the filtrates (5 mL) was fixed with 0.5 mL of HCl with a concentration of 50% (v/v), 0.5 mL of a metaphosphoric acid solution with a concentration of 187.5 g/L, and a solution of formic acid (250 mL), and then kept in a freezer at −18°C until the volatile fatty acid (VFA) and ammonia-N measurements. The VFA were determined using a gas chromatography (Agilent 6890 GC; Agilent Technologies, Wilmington, DE, USA) with a 30 m×0.25 mm×0.25 μm column (DB-FFAP, [17]) and ammonia N determined using a micro-Kjeldahl method (Kjeltec 8100; FOSS, Denmark [12,18]) were performed in quadruplicate, and the mean result was used for statistical analysis.

The second aliquot of filtrates (5 mL) was homogenized for microbiological detection and stored at −80°C until the relative abundances of rumen bacteria were determined. Preparation, extraction, and quantification of particular rumen bacteria DNA (including primers) were performed according to previous report [8]. References for the relative abundances of total bacteria, *Ruminococcus albus*, *Ruminococcus flavefaciens*, *Fibrobacter succinogenes*, *Butyrivibrio fibrisolvens*, *Megasphaera elsdenii*, *Streptococus bovis*, methanogen, and protozoa were purchased from Vivantis Technologies Sdn Bhd (Selangor Darul Ehsan, Malaysia). To establish uniformity, quantitative real-time polymerase chain reaction (PCR) experiments were performed in quadruplicate for each specified species or group of bacteria, using both standards and genomic DNA samples. The Ct data were converted into normalized relative numbers using the LightCycler 480 software version 1.2.9.11 (Basel, Switzerland), which was adjusted for PCR efficiency. The values for the 16S rRNA gene of a specific microorganism species or group are expressed as a percentage of total bacteria.

#### Carcass evaluation

The whole carcasses were immediately weighted after removing the non-carcass component to determine hot carcass weights. The whole carcasses were then chilled at 4°C for 48 h before being re-weighed to determine the cold carcass weights. The dressing percentage was calculated by dividing the hot carcass weight by the final BW and expressing the result as a percentage. Each carcass was then divided into left and right halves. Carcasses were cut up between the 12th and 13th ribs of the right halve to evaluate carcass quality. The 12th-rib fat thickness, longissimus muscle (LM) area (LMA), intramuscular fat, and final pH values of the carcass were measured. The carcass color was determined by measuring the L*, a*, and b* color values on the sliced lean surface and carcass external fat along the lateral side of the carcass with a portable Chroma Meter (CR-300; Minolta Corporation, Osaka, Japan). Color measurements were collected in quadruplicate in the CIELAB color spaces L* (0 = black, 100 = white), a* (negative values = green, positive values = red), and b* (negative values = blue, positive values = yellow), with considerable parts of connective tissue and intramuscular fat excised. Color saturation was determined using the previous reports [8,19].

The Warner-Bratzler shear force (WBSF) was determined in accordance with the AMSA (1995) standards, with minor adjustments [8]. After collecting data on carcass quality, strip loins were removed from the left side of each carcass, vacuum-packed, and stored to age at 4°C for 14 d. After aging, steaks with a thickness of 2.54 cm were prepared, vacuum-sealed, and frozen (−20°C) for subsequent examinations. WBSF steaks were defrosted at 4°C for 24 h before being cooked to an internal temperature of 70°C using an electric grill (Kashiwa KW-308; Nakhon Pathom, Thailand) fitted with thermocouples inserted about in the geometric center of the steak. The percentage of cooking loss was calculated by dividing the initial weight (before cooking) by the final weight (after cooking). Each steak was cut into at least six 1.27-cm-diameter pieces parallel to the muscle fiber orientation using a steel hollow-core tool [8]. The pieces were sheared perpendicular to the muscle fiber orientation using a shear device Texture evaluator for the Warner-Bratzler Meat Shear (TA-TX2 Texture Analyzer; Stable Micro Systems, Surrey, UK), set a crosshead speed of 200 mm/min and a 5 kN load cell calibrated to 113 over the range 0×100 N.

To assess ash, protein, moisture, and ether extractable lipid, external fat and connective tissue were removed from meat samples. The analyses were carried out in quadruplicate by following procedures described previously [8], and the mean data was used for statistical analysis.

### Statistical analysis

Analysis of data on animal performance, blood biochemical indexes, rumen fermentation and microbial community, as well as carcass characteristic were subjected to analysis of variance using the MIXED procedure of SAS 9.4 using the model: Y_ij_ = μ+τ_i_+ɛ_ij_, where Y_ij_ is the response variable, μ is the overall mean, τ_i_ is experimental diet (*i* = BS or TBS), and ɛ_ij_ is the residual error. The covariance structure was compound symmetry, which was opted in the SAS Kolmogorov-Smirnov test of the mixed model. The LSMEANS statement computes the treatment means after adjusting for the effect of the covariate. Animal performance and blood biochemical indices data were adjusted by incorporating the covariance mean as a covariable with initial sampling. The student’s t-test was used to evaluate all the data. The least-squares means were reported, and a significance level of p<0.05 was proclaimed.

## RESULTS

### Growth performance

The DMI, ADG, and the ADG and DMI ratio of the Thai-native Anglo-Nubian crossbred male goats are presented in Table 3. There was no difference in DMI, but the average final BW of goats fed TBS was 3% higher (p = 0.02) than their counterparts fed the BS diet, leading to a higher ADG/DMI for goats fed TBS diet.

### Rumen fermentation and microbial community

Rumen fluid pH values were not different between the two diets (averaged 6.8, Table 4). The concentration of ammonia N in the rumen was 1.1% lower (p = 0.01) while the overall VFA concentration was 2.2% higher (p = 0.03) for goats fed TBS as compared to goats fed BS diet. The substitution of TBS for BS resulted in a greater proportion of acetate (p = 0.01) and higher acetate to propionate ratio (p≥0.02). Propionate and butyrate proportions were not affected by feeding of TBS (Table 4).

**Figure 1:**
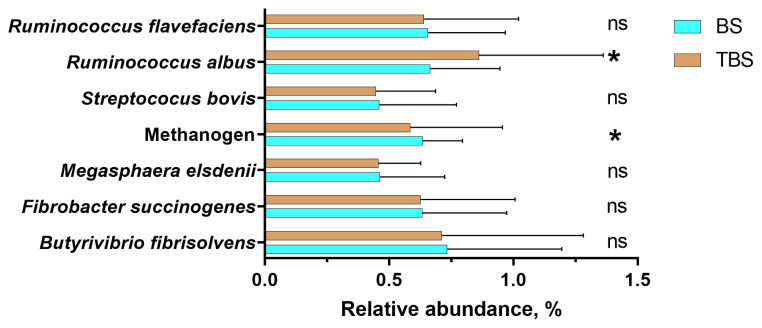
The relative abundances of selected microbial rumen bacteria in goats fed a total mixed ration supplemented with anthocyanin-rich black cane silage (BS) or anthocyanin-rich black cane silage treated with ferrous sulfate and molasses (TBS). The values are means, with horizontal bars representing standard errors. * p<0.05 indicating significant difference between treatment means; ns p>0.05 indicating no significant difference.

Results of the rumen microbial population showed that the abundances of total bacteria and total protozoa in the rumen fluid of goats fed the two diets were not significantly different. In addition, the relative abundances of *R. flavefaciens*, *F. succinogenes*, *B. fibrisolvens*, *M. elsdenii*, and *S. bovis* (Figure 1), expressed as percentage of the 16S rRNA gene copy number of the total bacteria (%), were also not different between the two groups. However, rumen fluid of goats fed TBS diet had 7.7% lower (p = 0.01) abundance of methanogens and 29.4% higher (p = 0.03) abundance of *R. albus* as compared to those fed BS diet.

### Blood biochemical indices

Plasma glucose, albumin, cholesterol, insulin, triglycerides, high density lipoprotein, low density lipoprotein, very low-density lipoprotein, and urea nitrogen were not affected by feeding of the TBS diet (Table 5). There were also no significant differences between the two dietary groups for concentrations of immunoglobin G, alanine transaminase, or aspartate aminotransferase. The concentration of TBARS was nearly 11% lower (p = 0.03) when the goats were fed TBS, but goats fed TBS diet resulted in increased plasma TAC concentration (p = 0.01) and higher activities of SOD, CAT, GSH-Px, and GSH-Rx (p = 0.01).

### Carcass and meat characteristics

Even though the final BW were significantly different (p<0.05), the hot and cold carcass weights and dressing percentage were not different between the two dietary groups (Table 6). TBS diet had no effect on the thickness of the 12th rib, the LMA, or the pH. There were also no significant differences in the indices of external fat color or 12th rib lean color between the two dietary groups. Cooking rate, drip loss, moisture, protein, and ash percentages of steaks were also not different between the two experimental diets (Table 7). The percentage of intramuscular fat in the steak samples was 20% higher (p = 0.01) in goats fed TBS diet, and their WBSF value of tenderness was found to be 11.4% lower (p = 0.01).

## DISCUSSION

To the best of our knowledge, the current study provides the first *in vivo* results on the feeding of FS-MS treated anthocyanin-rich black cane silage-based diet in ruminants. The results showed that treating anthocyanin-rich black cane silage with 0.03% FS and 4% MS (TBS) resulted in a relatively higher degradation of lignin and possibly its silica content. Most likely, the function of FS during biomass fermentation was to enhance the breakdown of lignin, hemicellulose, and cellulose during ensiling [1,5]. This was often a highly dehydrating process. In addition, MS appeared to enhance the FS catalytic process by increasing the moisture and soluble carbohydrate (WSC) content during the silage process, which promotes the growth of anaerobic and lactic acid bacteria [5].

Results of the current study showed that feeding a TMR diet containing 50% TBS to Thai-native×Anglo-Nubian male goats increased their ADG and feed conversion efficiency. The improvement in feed conversion efficiency observed in goats fed TBS is consistent with the fact that TBS diet significantly increased (p = 0.04) total rumen VFA concentration as compared with those fed untreated BS diet which is in agreement with earlier *in vitro* studies that FS-MS-induced VFA production [1,5].

The use of FS-MS to treat anthocyanin-rich black cane (TBS) could be a viable strategy to reduce anthocyanin loss during fermentation (Table 2). We postulate that the higher dietary anthocyanins because of feeding TBS improved total VFA concentration because anthocyanins play a regulatory role in growth of bacterial populations in the rumen as demonstrated by the increased relative abundance of *R. albus* in this study. It was indicated earlier that FS could hasten the breakdown of the lignin part in the TBS, which would result in an increase in the availability of sugar and, therefore, an increased production of fermentation acids for *R. albus* [1]. Although the nutrient digestibility of the diets was not determined, the higher relative abundance of *R. albus* in goats fed TBS diet appeared to support this notion. Others [20] reported that incorporating *Andrographis paniculata* leaves rich in plant active compounds (such as lactones, anthocyanin, flavonoids, and sterols) into goat diets enhanced ruminal *R. albus* population without altering the total bacteria which eventually led to an improvement in nutrient digestibility.

Our results reaffirmed a previous *in vitro* study [5] that black cane treated with 0.03% FS and 4% MS increased relative abundance of *R. albus* but no other bacteria, including *R. flavefaciens*, *F. succinogenes*, *B. fibrisolvens*, *M. elsdenii*, and *S. bovis*. The above findings also concord with those reported using black cane silage [8] and purple maize anthocyanin [9].

The lower ruminal ammonia-N concentrations in goats fed TBS diet deserves some mentioning. The above could be because TBS provided more fermentable carbohydrates which play an important role in increasing rumen microbes by converting rumen degradable CP to microbial protein [21,22]. However, it cannot exclude the possibility that greater anthocyanin consumption decreased rumen solubility of dietary proteins, which in turn decreased rumen ammonia-N concentrations [9]. Also, the above result could be affected by the 24 h fasting before slaughtering and collection of rumen fluid [16] used in this study. After 24 h of fasting, rumen fermentation may be quite mild and not reflecting its true potential [16]. It is unfortunate that the present study did not provide data to explain for anthocyanin induced protein solubility reduction, thus more research is needed on this interesting subject. In addition, feeding TBS diet resulted in a decrease in the relative quantity of methanogens in the rumen fluid sample. In fact, both BS or TBS diet contained anthocyanin components. The result of anthocyanin reducing methanogens in presence of higher *R. albus* population observed in this study is rather difficult to explain, but similar observations of reduced relative abundance of methanogens in rumen fluids in sheep and goats after feeding mulberry leaf [23] and black cane [8] had been reported. Perhaps, the reduced relative abundance of methanogens in TBS goats could be explained by the higher anthocyanin components and FS residue in the rumen fluid, which might inhibit methanogenesis by making electron exchange more challenging for FS-reducing bacteria and methanogens harboring Fe oxides [24]. This hypothesis, however, contradicts the increased rumen acetate to propionate ratio in TBS fed goats in this study. The current finding might be read as feeding of TBS diet reduced methane production via reductive acetogenesis [25], because synthesis of acetate, as opposed to the production of propionate, is commonly acknowledged as a source of hydrogen [25,26]. It is difficult to explain the apparent difference between present acetic acid results and the relative abundance of methanogens, but it cannot be ruled out that acetogenic bacteria used hydrogen to produce acetate. Acetogenic bacteria can, in fact, transform carbon dioxide and hydrogen straight into acetate [27]. Further research is required to shed additional light on this subject.

Feeding of TBS had no effect on any of the carcass param eters investigated (e.g., dressing percentage, LMA, and pH value) in this study, which is in line with other reports in small ruminants [8,19]. However, feeding of TBS to cattle resulted in favorable effect, including external fat color [28]. The discrepancy in the results among studies could be attributed to a number of factors, including the composition of the anthocyanin-based diets, the animals and the bioavailability of flavonoids or anthocyanins. Also, neither the adipose nor the lean tissue color (L*, a*, b*, or c*) of the goats was influenced by diets. Anthocyanins containing malvidin, malvidin-3-O-glucoside, and peonidin-3-O-glucoside were found to be capable of stabilizing muscle membranes, resulting in improved meat color [8,19]. In this study, both the BS and TBS diets contained peonidin-3-O-glucoside, malvidin-3-O-glucoside, and malvidin, which may have reduced lipid peroxidation and preserve meat coloration. Our results showed that TBS diet promotes higher intramuscular fat content. The increased intramuscular fat accumulation in the TBS-fed goat muscles could most likely due to the increased rumen acetate synthesis as increasing acetate production opens up additional substrate for *de novo* fat synthesis [10]. Previous research [29] reported a link between intramuscular fat content and WBSF tenderness values. Therefore, it is postulated that the lower WBSF values obtained in current study are, at least in part, attributable to greater intramuscular fat concentrations.

Blood biochemical indices can be useful indicators for animal’s health status relating to dietary absorption and metabolism [7]. We hypothesized that the anthocyanins and other bioactive compounds in the TBS diet can help to reduce oxidative stress in animals (goats in this case) under stress in the tropics. Results of the blood biochemical analysis in this study, specifically, the lower TBARS values and higher TAC levels supported the above. Similar findings were also reported involving anthocyanin in purple maize [9], mangosteen peel [7], and piper meal [6]. It is believed that the higher level of anthocyanins (such as peonidin-3-O-glucoside, malvidin-3-O-glucoside, and malvidin) provided better protect against free radicals and decrease inflammation [8] in the TBS fed goats. Simultaneously, feeding TBS resulted in an increase in the activity of antioxidant enzymes such as SOD, CAT, and GSH. In this aspect, our results concord with previous study from this laboratory [6] that lactating goats fed a mixed diet that included piper meal rich in flavonoids (possibly anthocyanin as well), essential oils, and phenolic acids altered oxidative markers (SOD, CAT, and GSH) activities in the rumen, blood, milk, and mammary tissue. Increasing antioxidant enzyme activity in ruminal fluid or blood may result in a decrease in TBARS levels by optimizing the rumen and its rumen bilayers for dietary bioactive chemicals produced from piper meal by increasing membrane fluidity [30]. Based on the current results, we postulated that the higher level of anthocyanin in the TBS diet acts as an electron donor, reducing the accumulation of reactive oxygen species (ROS), as SOD is the first line of defense against ROS scavenging in intracellular enzymes. In contrast, activation of CAT and GSH-Px or GSH-Rx implies that the superoxide anion radical in the dismutation exceeded the limit, resulting in the ROS being scavenged enzymatically by CAT or GSH before being reduced to water [8]. It appeared that SOD, CAT, and GSH were all acting together to reduce ROS scavenging. Therefore, the lower TBARS values and the higher TAC levels are not only induced by the antioxidative activity of anthocyanin, but also by the greater expressions of SOD, CAT, and either GSH-Px or GSH-Rx.

## CONCLUSION

Feeding TMR containing 50% anthocyanin-rich black cane silage treated with 0.03% ferrous sulfate and 4% molasses significantly enhanced growth rate and feed conversion efficiency in goats. This enhancement in animal productivity was partly due to higher rumen fermentation activity, as evident by the higher total VFA production and the relative abundance of cellulolytic bacteria, *R. albus* due to the higher level of anthocyanin in the TBS diet. Concurrently, the higher anthocyanin content of the TBS diet reduced stress by increasing antioxidant activity, as evidenced by lower TBARS concentrations but higher TAC, SOD, CAT, GSH-Px, and GSH-Rx concentrations in blood plasma, resulting in improved health. Feeding TBS, which contained higher anthocyanin, also resulted in higher fat content and more tender meat in goats. Goats fed the TBS diet had lower rumen ammonia-N and relative abundance of methanogens in the presence of the higher acetic/propionic ratio; however, these phenomena require further investigation. Collectively, the findings of this study could provide insights into the nature of hydrolytic pretreatments for lignocellulose agricultural biomass and roughage-based diets as their health-improving features can provide a viable alternative to the use of antibiotics and synthetic growth promoters in the production of sustainable animal feed and animal products.

## Figures and Tables

**Table 1:** Chemical composition and fermentation characteristic of BS and TBS

Item	Treatment^1)^	

	BS	TBS
Chemical composition (% dry matter basis, unless otherwise stated)		
Dry matter (% fresh weight basis)	16.00	15.7
Crude protein	7.3	7.6
Neutral detergent fiber	79.2	79.1
Acid detergent fiber	49.2	49.0
Neutral detergent lignin	6.1	5.6
Hemicellulose	30.0	30.1
Cellulose	43.1	43.5
Ash	13.8	13.9
Fermentation characteristic (% dry matter basis)		
pH value	4.8	3.7
Lactic acid	3.5	8.1
Acetic acid	2.7	2.9
Propionic acid	-	-
Butyric acid	-	-
Ammonia nitrogen	0.03	0.03

1) BS, anthocyanin-rich black cane silage; TBS, anthocyanin-rich black cane silage treated with ferrous sulfate heptahydrate and molasses.

**Table 2:** Ingredients and nutrient composition of experimental diets

Item	Treatment^1)^	

	BS	TBS
Ingredient, dry matter basis		
Anthocyanin-rich black cane silage	50.0	-
Treated anthocyanin-rich black cane silage^2)^	-	50.0
Cassava pulp	3.8	3.8
Cassava chip	19.5	19.5
Soybean meal	4.0	4.0
Rice bran	5.5	5.5
Palm meal	14.0	14.0
Mineral mix^3)^	0.7	0.7
Compound premix^4)^	0.2	0.2
Sunflower oil	1.0	1.0
Sulfur^5)^	0.4	0.4
Urea	0.9	0.9
Nutrient composition, dry matter basis		
Metabolizable energy (Mcal/kg)	4.8	4.8
Crude protein (%)	12.1	12.2
Neutral detergent fiber (%)	67.0	66.9
Acid detergent fiber (%)	33.2	33.1
Neutral detergent lignin (%)	4.5	4.2
Hemicellulose (%)	33.8	33.8
Cellulose (%)	28.8	28.9
Ash (%)	11.8	11.8
Total anthocyanins (mg/g)	0.17	0.75
Cyanidin-3-glucoside	0.01	0.02
Pelargonidin-3-glucoside	0.01	0.04
Delphinidin	0.03	0.10
Peonidin-3-O-glucoside	0.03	0.13
Malvidin-3-O-glucoside	0.02	0.13
Cyanidin	0.04	0.19
Pelargonidin	0.003	0.01
Malvidin	0.02	0.13

1) BS, anthocyanin-rich black cane silage; TBS, anthocyanin-rich black cane silage treated with ferrous sulfate heptahydrate and molasses.

2) Anthocyanin-rich black cane treated with 4% of molasses and 0.03% of ferrous sulfate heptahydrate.

3) Contained (g/kg): NaCl (600), P (160), Ca (240).

4) Vitamin A (4,200.000 IU/kg), vitamin A_3_ (840,000 IU/kg), vitamin E (10,000 IU/kg), vitamin K_3_ (2 g/kg), vitamin B_1_ (2.4 g/kg), vitamin B_2_ (3.5 g/kg), vitamin B_6_ (1.8 g/kg), vitamin B_12_ (0.01 g/kg), vitamin B_5_ (4.6 g/kg), vitamin C (12 g/kg), folic acid (0.28 g/kg), vitamin 7 (0.4 g/kg), copper (12 g/kg), manganese (40 g/kg), zinc (3.2 g/kg), iron (42 g/kg), iodine (0.8 g/kg), cobalt (0.8 g/kg), selenium (0.35 g/kg).

5) Sulfur cube was derived from commercial purchase (Sand Sea Sun Shop: TG-6731, Bangkok, Thailand) and ground (sieve size of 1 mm).

**Table 3:** Growth performance of Thai-native×Anglo-Nubian crossbred male goats fed a total mixed ration supplemented with BS or TBS

Item	Experimental diet^1)^		SEM	p-value

	BS	TBS		
Animal number	16	16	-	-
Initial body weight (kg)	14.5	14.5	0.580	0.511
Final body weight (kg)	17.8	18.4	0.833	0.020
ADG (g/d)	36.5	43.6	0.829	0.040
DMI (g/d)	458.7	484.9	0.941	0.316
ADG/DMI	0.08	0.09	0.860	0.041

SEM, standard error of mean; ADG, average daily gain; DMI, dry matter intake.

1) BS, anthocyanin-rich black cane silage; TBS, anthocyanin-rich black cane silage treated with ferrous sulfate heptahydrate and molasses.

**Table 4:** Rumen fermentation of Thai-native×Anglo-Nubian crossbred male goats fed a total mixed ration supplemented with BS or TBS

Item	Experimental diet^1)^		SEM	p-value

	BS	TBS		
pH value	6.9	6.7	0.209	0.681
Ammonia N (mg/dL)	13.3	13.2	0.488	0.011
Total VFAs (mM)	84.5	86.3	0.901	0.035
Individual VFA (molar % of total VFAs)				
Acetate	59.9	61.0	0.730	0.001
Propionate	27.9	27.2	0.580	0.120
Butyrate	12.1	11.8	0.605	0.190
Acetate/propionate	2.1	2.2	0.116	0.027

SEM, standard error of mean; VFAs, volatile fatty acids.

1) BS, anthocyanin-rich black cane silage; TBS, anthocyanin-rich black cane silage treated with ferrous sulfate heptahydrate and molasses.

**Table 5:** Blood biochemical indices of Thai-native×Anglo-Nubian crossbred male goats fed a total mixed ration supplemented with BS or TBS

Item	Experimental diet^1)^		SEM	p-value

	BS	TBS		
Total protein (g/L)	72.4	72.1	0.962	0.575
Albumin (g/L)	33.3	33.2	0.764	0.166
Globulin (g/L)	39.1	38.9	0.691	0.253
Blood urea N (mmol/L)	8.0	8.0	0.314	0.879
Insulin (μU/mL)	1.7	1.7	0.440	0.194
Glucose (mmol/L)	5.1	5.2	0.468	0.596
Total cholesterol (mmol/L)	3.1	3.0	0.896	0.138
Triglyceride (mmol/L)	0.6	0.5	0.136	0.108
HDL (mmol/L)	1.7	1.8	0.504	0.085
LDL (mmol/L)	1.0	1.0	0.361	0.069
VLDL (mmol/L)	1.1	1.1	0.496	0.171
Alanine transaminase (U/L)	29.8	29.8	0.616	0.362
Aspartate aminotransferase (U/L)	35.0	35.0	0.670	0.410
IgG (g/L)	12.1	12.1	0.487	0.834
TAC (nmol/μL)	32.6	40.1	0.770	0.004
SOD (U/mL)	83.8	93.5	0.666	0.002
CAT (U/mL)	68.2	75.9	0.696	0.017
GSH-Px (U/mL)	44.0	48.8	0.339	0.030
GSH-Rx (U/mL)	53.2	63.7	0.468	0.022
TBARS (nmol/mL)	40.2	36.0	0.444	0.034

SEM, standard error of mean; HDL, high density lipoprotein; LDL, low density lipoprotein; VLDL, very low-density lipoprotein; IgG, immunoglobin G; TAC, total antioxidant capacity; SOD, superoxide dismutase; CAT, catalase; GSH-Px, glutathione peroxidase; GSH-Rx, glutathione reductase; TBARS, thiobarbituric acid-reactive substances.

1) BS, anthocyanin-rich black cane silage; TBS, anthocyanin-rich black cane silage treated with ferrous sulfate heptahydrate and molasses.

**Table 6:** Carcass characteristics of Thai-native × Anglo-Nubian crossbred male goats fed a total mixed ration supplemented with BS or TBS

Item	Experimental diet^1)^		SEM	p-value

	BS	TBS		
BW (kg)	17.8	18.4	0.833	0.019
HCW (kg)	8.5	8.88	0.570	0.349
CCW (kg)	8.4	8.7	0.828	0.269
DP (%)	48.0	48.1	0.746	0.110
12th – rib fat thickness (cm)	0.2	0.2	0.040	0.634
LMA (cm^2^)	14.5	14.5	0.544	0.136
pH value	6.8	6.8	0.419	0.072
External fat color^2),3)^				
L*	22.4	22.5	0.603	0.569
a*	15.2	14.9	0.404	0.194
b*	6.6	6.7	0.339	0.168
c*	16.6	16.3	0.439	0.649
12th – rib lean color				
L*	48.7	48.8	0.439	0.158
a*	9.9	9.8	0.310	0.140
b*	6.8	6.9	0.390	0.204
c*	12.0	11.9	0.393	0.190

SEM, standard error of mean; BW, body weight; HCW, hot carcass weight; CCW, cold carcass weight; DP, dressing percentage; LMA, LM area measured at 12th rib.

1) BS, anthocyanin-rich black cane silage; TBS, anthocyanin-rich black cane silage treated with ferrous sulfate heptahydrate and molasses.

2) Fat color measurements obtained approximately 20 cm ventrally to the lateral process of the split carcass adjacent the 13th rib.

3) CIE color measurements: L* = lightness, black (0) to white (100); positive a* = red; negative a* = green; positive b* = yellow; negative b* = blue; c* = color saturation = [(a*)^2^+(b*)^2^]^1/2^ whereby a large number is considered to be more vivid.

**Table 7:** WBSF and chemical composition of steak samples of Thai-native×Anglo-Nubian crossbred male goats fed a total mixed ration supplemented with BS or TBS

Item	Experimental diet^1)^		SEM	p-value

	BS	TBS		
WBSF (kg)	7.5	6.6	0.271	0.001
Cooking rate (%)	43.0	42.6	0.335	0.426
Drip loss (%)	8.7	11.5	0.465	0.219
Moisture (%)	79.3	79.2	0.237	0.750
Protein (% DM)	88.4	88.4	0.550	0.835
Intramuscular fat (% DM)	5.6	6.8	0.254	0.004
Ash (% DM)	6.2	6.4	0.450	0.117

WBSF, Warner-Bratzler shear force values of tenderness; SEM, standard error of mean; DM, dry matter.

1) BS, anthocyanin-rich black cane silage; TBS, anthocyanin-rich black cane silage treated with ferrous sulfate heptahydrate and molasses.
